# Supplementary material for: Addressing human-tiger conflict using socio-ecological information on tolerance and risk
Source: Nat Commun. 2018 Aug 27;9:3455. doi: 10.1038/s41467-018-05983-y (PMC6110717; doi:10.1038/s41467-018-05983-y)
Supplement: Supplementary file 1 — Supplementary Information [file 41467_2018_5983_MOESM1_ESM.pdf]

Addressing human-tiger conflict using socio-ecological information on tolerance and risk

Struebig et al. Nature Communications, 2018.

Supplementary Information

Supplementary Note 1

Landscape Connectivity for Tigers. We used the algorithm Circuitscape (www.circuitscape.org[1]) to predict patterns of tiger connectivity across the forest-farmland interface from key landscape features. The algorithm uses electronic circuit and random walk theories to simulate animal movements across areas of variable resistance, and typically outperforms other connectivity models [2]. The most important landscape variables deemed to influence tiger movements in human-dominated habitats were land-cover, topography and watercourses. Land-cover influences prey densities and shelter, and is known to be an important predictor of tiger movement across the species range [3]. In Kerinci Seblat tigers are seen in all forest types and smallholder tree plantations, but rarely reported in open farmland or large-scale plantations. Occupancy appears to be independent of elevation, but detections from cameras tend to be higher in flat areas compared to rugged terrain[4]. Finally, tigers are able to cross small rivers, but translocated tigers are also known to follow watercourses in lowland areas [5]. We therefore integrated this information as a landscape resistance surface according to the layers and values in Supplementary Table 1, so that forest-like vegetation had lower resistance at any elevation, and that riparian areas would have reduced resistance (less 10) in rugged areas, but not in flat areas (i.e. to prioritise crossing a river in rugged areas, but following a river in the lowlands). The Circuitscape model defined core forest blocks as tiger sources, and treated small forest patches (< 3km^2) as part of the landscape matrix. The resulting layer highlighted farmland areas completely surrounded by the national

park to be particularly important for tiger movement, whereas open areas away from core forest blocks were less connected (Supplementary Figure 1).

Supplementary Table 1. Landscape variables explored as potential predictors in models of human-tiger encounter around Kerinci Seblat, Sumatra, Indonesia.

Variable	Variable contribution to ensemble model of risk	Source and information
Distance to rivers (km)<br>Two variants - small, or large – based on catchment sizes	0.05	- Watercourses defined from digital elevation model, http://srtm.csi.cgiar.org
Distance to forest (km)	0.06	- Forest cover in 2010 [6]
Tiger connectivity (Amps)	0.06	- Derived by circuitscape modelling based on a resistance surface, which combined information from land-cover and topography (see Supplementary Table 2), and rivers.
Human population density (people per km^2)	0.07	- Landscan 2012. http://web.ornl.gov/sci/landscan/
Tiger occupancy, Ψ	0.09	- Based on tiger surveys implemented between 2007 and 2009 [7], spatially interpolated to non-surveyed areas.
Percent forest cover	0.13	- Forest cover in 2010 [6], calculated within 3.25km radius of a point.
Distance to roads (km)	0.16	- Road layer from Indonesian National Coordination Agency for Surveys and Mapping for Sumatra [4]
Farmer density (people per km^2)	0.38	- Bureau of Statistic (BPS) Indonesia. Jakarta, Indonesia, https://microdata.bps.go.id/mikrodata/index.php/catalog/SUSENAS (2010).<br>- Mapped to village administrative boundaries, incorporating boundary and name changes in 2009. Defined by livelihoods: farm, garden, fish livestock and other farm.

Variable contribution was calculated as a Pearson coefficient of the correlation between fitted values and predictions in which the target variable was permutated via a randomisation procedure [8]. Low values indicate low correlation, and hence strong variable contribution to final model.

Supplementary Table 2. Landscape variables used to create a resistance surface on which tiger connectivity was derived around Kerinci Seblat, Sumatra, Indonesia.

**Variable**	**Source and information**
2010 land-cover, in 15 classes (codes from source; tiger resistance values in parentheses):<br>1. Water (100)<br>3. Swamp forest (20)<br>4. Lowland forest (1)<br>5. Lower montane forest (1)<br>6. Upper montane forest (1)<br>7. Plantation/regrowth (50)<br>8. Lowland mosaic (80)<br>9. Montane mosaic (80)<br>10. Lowland open (90)<br>11. Montane open (90)<br>12. Urban (100)<br>13. Large-scale oil palm (90)<br>14. Plantation/regrowth, deforested (80)<br>15. Lowland mosaic, deforested (50)<br>16. Montane mosaic, deforested (50)	- Based on MODIS data at 250 m resolution [9]. Lowland = 0-750 m.a.s.l; lower montane = 750-1500 m.a.s.l; upper montane >1500 m.a.s.l.<br>- Then integrated with forest loss since 2005 [6] to identify areas in the farmland/plantation mosaic with more open, degraded vegetation.
Topographic ruggedness<br>Reclassified to 'low', 'medium' and 'high' based on quartiles.	- Calculated from 90m resolution SRTM digital elevation model (http://srtm.csi.cgiar.org/): an index of average distance in elevation between a focal cell and its neighbours [10].
Distance to rivers (km)<br>Two variants - small, or large – based on catchment sizes	- Watercourses defined from digital elevation model, http://srtm.csi.cgiar.org

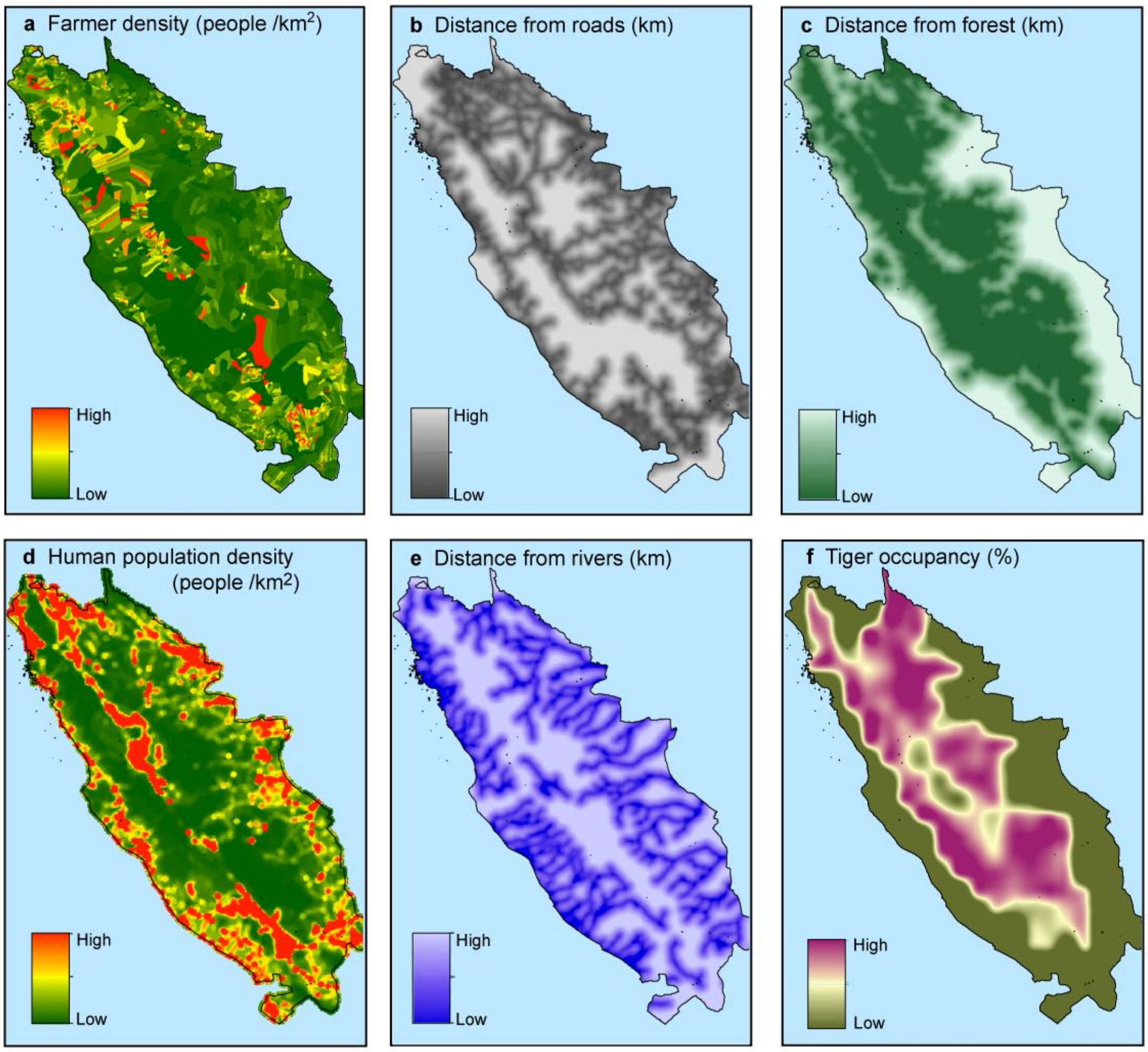

Supplementary Figure 1. Landscape covariates used in ensemble models of tiger encounter risk (a-f).

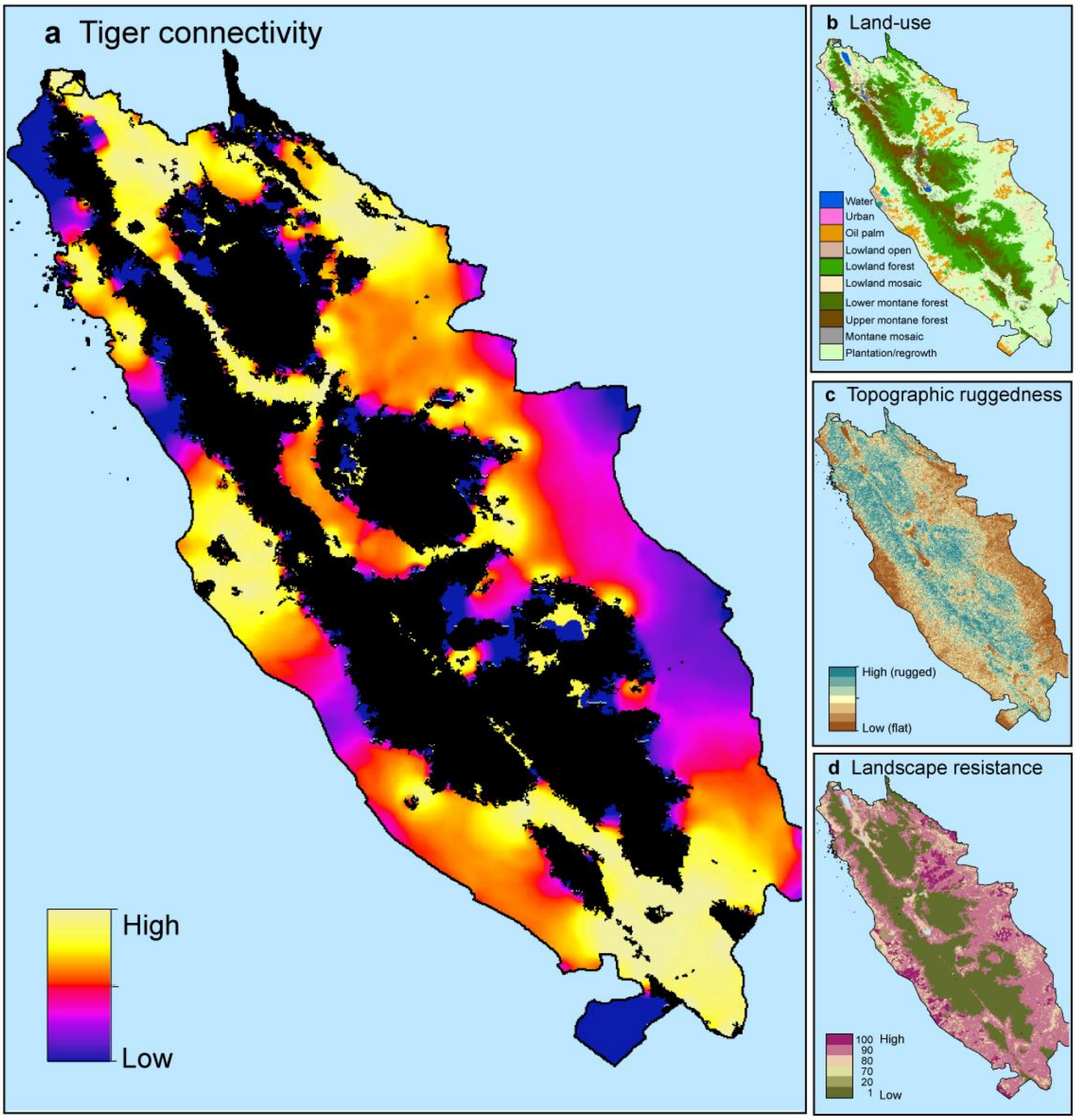

Supplementary Figure 2. Tiger connectivity (a), and the landscape covariates (b-c) used in addition to distance from rivers to generate a landscape resistance surface (d), on which circuitscape analyses were based.

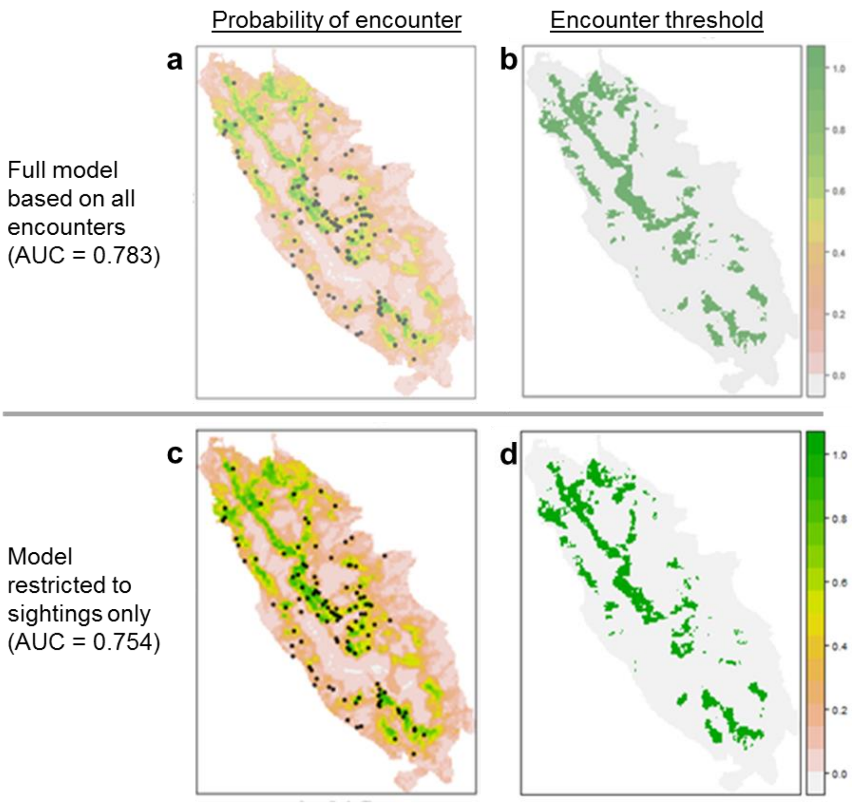

Supplementary Figure 3 Outputs from ensemble models that included all encounter data (a, b) or only sightings data (c,d). Maps show the probability of encounter for each ensemble model (a, c), and for visual comparison the predicted area of encounter as determined via a threshold based on the model true skills statistic.

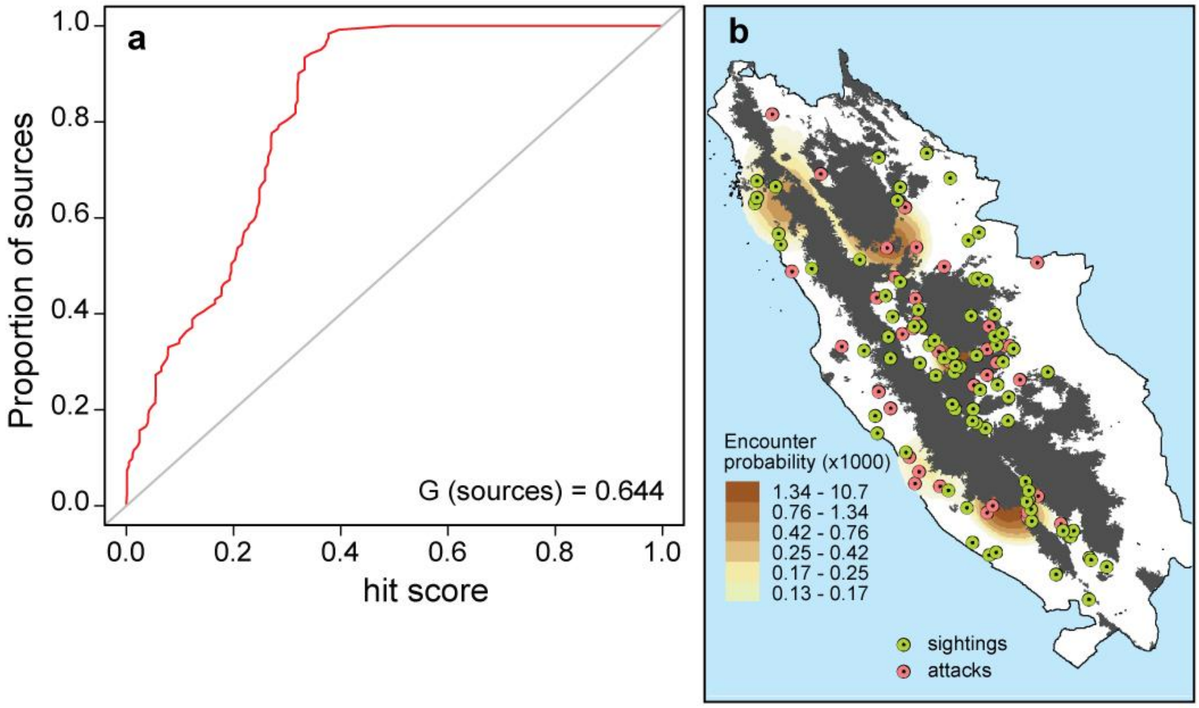

Supplementary Figure 4 Output from the geographic profiling model. (a) Gini plot showing the proportion of tiger encounter (sources) identified from the sightings data against hitscore (total area searched) within the geographic profile. (b) Geographic profile based on a subset of the encounter data, mapped together with source localities (sightings; black circles) and test locations (attacks on livestock, people and tigers; red squares). Contours show bands of 5%, with lighter colours corresponding to higher parts of the profile.

Supplementary Table 3. Questionnaire items used to measure tolerance for tigers in village surveys.

**Variable label**	**Item**
Tolerance[a] (response variable)	People's preference foe the size of the local tiger population: 'Do you think that the tiger population around here should be completely eradicated, reduced in number, kept the same or allowed to increase in number?'
Affect[b]	
BadGood	"Even if you have never seen this animal before, we are interested in what you immediately feel about it. Which number best describes your opinion of the animal on the picture card?"
DangHarm	"Even if you have never seen this animal before, we are interested in what you immediately feel about it. Which number best describes your opinion of the animal on the picture card?"
Attitude[c]	
Kill tiger	"These days I think that tigers in the village, on the farm land around the village and in the forest should be caught."
Protect tiger	"These days I think that tigers around here should be protected. By around here I mean in the village on the farm land around the village and in the forest."
Norms[c]	
Injunctive	"People around here want you (the men in your household) to catch tigers."
Descriptive	"Most people like you (like the men in your household) try to catch tigers around here."
Beliefs[c]	
Spirit	"It is beneficial to your spiritual wellbeing to have tiger around here."
Health	"It is beneficial to your health to have tiger around here."
Env	"It is detrimental to the health of the environment to have tigers around here."
Trust[c]	
TrustA	"I trust the national park and BKSDA people to make the right decision about how to manage the wild animals that live in this area."
TrustB	"I trust the national park and BKSDA people to keep people safe from any dangerous animals."
Management scenarios[d]	
ScenA	A tiger is seen in the forest-farm area posing no threat to people.
ScenB	A tiger is seen in the forest-farm area and is a threat to people.
ScenC	A tiger near the village has attacked livestock.
ScenD	A tiger near the village has attacked a person.
Demographics	
Age	"What year were you born?"
Ethnicity	"How would you describe your ethnic origin e.g. are you Sudanese, Javanese, Kerincinese, Minangkabau or mixed?"
Sex	Recoded directly by enumerator.

[a] Four-point scale: 'Completely eradicate', 'Reduce in number', 'Kept the same in number'. 'Don't know' was also permitted as a response. Prior to analysis, 'Completely eradicate' and 'Reduce in number' were collapse into one category with the variable treated as categorical thereafter. [b] Semantic scale with the words 'Good – Bad', 'Dangerous – Harmless' displayed at either end. [c] Five-point Likert response ('Strongly agree' to 'Strongly disagree') scaled from 1-5. [d] Five-point ordinal scale: 'Kill the tiger' = 1, 'Remove tiger from the wild' = 2, 'Move tiger to another forest area' = 3, 'Monitor situation' = 4, 'Do nothing' = 5.

Supplementary Table 4. Numbers and proportions of 75 villages ranked as low, medium or high priority for intervention using measures of human-tiger encounter risk and people's tolerance. The number of independent attacks on livestock or people, as well as tigers removed from these villages is also presented. Priority rank is based on the distribution of encounter risk and tolerance data: 'low' represents below median risk; medium is above median risk and above median tolerance; high is above median risk and below median tolerance. The role of tolerance data in the prioritisation can be deduced by comparing the villages classed as high versus medium risk.

	Priority					
**No. villages**	**Low**		**Medium**		**High**	
**a** using ensemble risk model						
Villages with encounters	8	(11%)	32	(44%)	32	(44%)
Attacks on livestock or people	4	(5%)	30	(41%)	40	(54%)
Tigers removed	3	13%	5	(22%)	15	(65%)
**b** using geographic profile						
Villages with encounters	63	(88%)	3	(4%)	6	(8%)
Attacks on livestock or people	62	(84%)	1	(1%)	11	(15%)
Tigers removed	15	(65%)	0	(0%)	8	(35%)

SUPPLEMENTARY REFERENCES

1 McRae, B. H., Dickson, B. G., Keitt, T. H. & Shah, V. B. Using circuit theory to model connectivity in ecology, evolution and conservation. *Ecology* **89**, 2712-2724, doi:10.1890/07-1861.1 (2008).

2 McRae, B. H. & Beier, P. Circuit theory predicts gene flow in plant and animal populations. *Proc. Natl Acad. Sci. USA* **104**, 19885-19890 (2007).

3 Wikramanayake, E. *et al.* Designing a conservation landscape for tigers in human-dominated environments. *Conserv. Biol.* **18**, 839-844 (2004).

4 Linkie, M. *et al.* Safeguarding Sumatran tigers: evaluating effectiveness of law enforcement patrols and local informant networks. *J. Appl. Ecol.* **52**, 851-860 (2015).

5 Priatna, D., Santosa, Y., Prasetyo, L. B. & Kartono, A. P. Home range and movements of male translocated problem tigers in Sumatra. *Asian J. Conserv. Biol.* **1**, 20-30 (2012).

6 Margono, B. A., Potapov, P. V., Turubanova, S., Stolle, F. & Hansen, M. C. Primary forest cover loss in Indonesia over 2000-2012. *Nature Clim. Change* **4**, 730-735 (2014).

7 Wibisono, H. T. *et al.* Population status of a cryptic top predator: an island-wide assessment of tigers in Sumatran rainforests. *PLoS ONE* **6**, e25931 (2011).

8 Thuiller, W., Lafourcade, B., Engler, R. & Araújo, M. B. BIOMOD – a platform for ensemble forecasting of species distributions. *Ecography* **32**, 369-373 (2009).

9 Miettinen, J., Shi, C., Tan, W. J. & Liew, S. C. 2010 land cover map of insular Southeast Asia in 250-m spatial resolution. *Remote Sensing Letters* **3**, 11-20 (2011).

10 Wilson, M. F. J., O'Connell, B., Brown, C., Guinan, J. C. & Grehan, A. J. Multiscale terrain analysis of multibeam bathymetry data for habitat mapping on the continental slope. *Marine Geodesy* **30**, 3-35 (2007).
